# An unusually high frequency of SCAD deficiency caused by two pathogenic variants in the *ACADS* gene and its relationship to the ethnic structure in Slovakia

**DOI:** 10.1186/s12881-018-0566-0

**Published:** 2018-04-20

**Authors:** Jana Lisyová, Ján Chandoga, Petra Jungová, Marcel Repiský, Mária Knapková, Martina Machková, Svetozár Dluholucký, Darina Behúlová, Jana Šaligová, Ľudmila Potočňáková, Miroslava Lysinová, Daniel Böhmer

**Affiliations:** 1 Institute of Medical Biology, Genetics and Clinical Genetics, Faculty of Medicine, Comenius University and University Hospital, Department of Molecular and Biochemical Genetics - Centre of Expertise for Molecular and Biochemical Genetics of Rare Diseases, Bratislava, Slovakia; 2 Newborn Screening Centre of SR, Children’s Faculty Hospital, Banská Bystrica, Slovakia; 3 Department of Laboratory Medicine, University Children’s Hospital, Bratislava, Slovakia; 4 Metabolic Clinic, Children’s Faculty Hospital, Košice, Slovakia; 5 Second Paediatric Department of Slovak Medical University, Children’s Faculty Hospital, Banská Bystrica, Slovakia

**Keywords:** Short-chain acyl-CoA dehydrogenase deficiency, Newborn screening, C4-acylcarnitine, Ethylmalonic acid, Frequent pathogenic variants in Slovakia, Roma ethnic group

## Abstract

**Background:**

Short-chain acyl-CoA dehydrogenase deficiency (SCADD) represents a rare autosomal recessive inborn metabolic disorder of mitochondrial β-oxidation of monocarboxylic acids. Clinical symptoms can vary from a severe life-threatening condition to an asymptomatic state, reported in the majority of cases. Since the expansion of newborn screenings, more than three hundred probands were admitted for molecular-genetic analysis, most selected because of elevated values of C4-acylcarnitine detected in newborn screenings in Slovakia. Searching for the principal genomic changes led us to the selection of sixty-two patients in whom the presence of sequence variants in the *ACADS* gene was analysed and correlated with the available biochemical and clinical data.

**Methods:**

Biochemical and molecular genetic tests were performed. Acylcarnitine profiles focused on an elevated level of C4-acylcarnitine, which was analysed via tandem mass spectrometry. Urinary organic acids, specifically a quantity of ethylmalonic acid, were determined by gas chromatography/mass spectrometry. The entire coding region of the *ACADS* gene was sequenced. A low-cost restriction fragment length polymorphism of PCR amplified fragments analysis (PCR-RFLP) of pathogenic variants was introduced and implemented for the molecular-genetic algorithm appropriate for the Slovak population.

**Results:**

Our molecular genetic study was performed on sixty-two patients with a pathological biochemical pattern related to short-chain acyl-CoA dehydrogenase deficiency. In this cohort, we discovered a high occurrence of two rare pathogenic variants—the deletion c.310_312delGAG and the substitution c.1138C>T, with allelic frequencies of 64% and 31%, respectively. Up to 86% of investigated individuals belong to the Roma ethnic group.

**Conclusions:**

Analogous to other countries, SCADD is not included in the newborn screening programme. Based on the exceeded levels of the specific biomarker C4-acylcarnitine as well as ethylmalonic acid, we revealed a high prevalence of short-chain acyl-CoA dehydrogenase deficiency cases, confirmed by the findings of two rare pathogenic variants. A deletion c.310_312delGAG and c.1138C > T substitution in the *ACADS* gene appear with a high frequency in the Roma ethnic group of Slovakia. Due to the uncertainty of the pathogenicity and clinical consequences, it is important to follow up the morbidity and mortality in these patients over time and evaluate SCADD in relation to clinical outcomes and preventive healthcare recommendations.

## Background

Short-chain acyl-CoA dehydrogenase deficiency (SCADD, OMIM #201470) is a rare inborn metabolic disorder of mitochondrial β-oxidation with autosomal recessive inheritance. It was first described by Turnbull et al. [1] in a patient with lipid storage myopathy and low concentrations of carnitine and soon after by Bennett et al. [2] in a patient with defective oxidation of butyrate and hexanoate and excessive excretion of ethylmalonic acid in urine. Subsequently, SCADD was reported in two unrelated neonates with metabolic acidosis and ethylmalonate aciduria by Amendt et al. [3]. Based on the available data, clinical presentation can range from the severe infantile form with metabolic acidosis and neurological impairment to an asymptomatic state [4]. A manifestation of the deficiency may occur in stressful situations such as prolonged starvation, physical load, and infection or after an intake of food rich in fats. However, the majority of SCAD-deficient children detected by a newborn screening are asymptomatic at the time of diagnosis, and most of them remain asymptomatic during the long period of observation. Based on the published data, the prevalence ranges from 1 in 35,000 to 1 in 50,000 [4]. According to data acquired from Slovak newborn screenings in 2016, the prevalence of SCADD in Caucasian newborns is 1:9.745, and in Roma newborns, it is very high, with as many as 1:100.

The metabolic presentation of mitochondrial β-oxidation defects is based on the role of fatty acids in energy supplementation, mainly during prolonged fasting and resulting catabolism. The specific mitochondrial enzyme—short-chain acyl-CoA dehydrogenase (SCAD, EC 1.3.8.1.)—catalyses the first step in the mitochondrial β-oxidation of monocarboxylic acids four to six carbons in length. SCAD belongs to the flavoenzymes, and the final complex represents a homotetramer, functioning with FAD as a cofactor. Because butyryl-CoA (C4-CoA) represents the favoured enzyme substrate, the presence of deficient variants of this protein is associated mainly with the elevation of C4-CoA by-products derived from alternative metabolic routes of the accumulated C4-CoA—butyrylcarnitine, butyrylglycine, butyrate, ethylmalonic acid (EMA), and methylsuccinic acid in blood, urine and some types of cells [5, 6]. To compare the pathobiochemical role of SCAD, it is important to stress that acyl-CoA dehydrogenases (MCAD, LCHAD) play an important role in the production of acetyl-CoA and ketone bodies during prolonged starvation in the liver. However, because SCAD activities do not have an important effect on acetyl-CoA production and ketone bodies synthesis, nonketotic hypoglycaemia is ordinarily not present in SCADD.

At the molecular-genetic level, SCADD is due to the presence of pathogenic variants in the *ACADS* gene (12q24.31), which spans approximately 13 kb and consists of ten exons [7]. To date, according to the Human Gene Mutation Database Professional (HGMD® Professional), eighty-five pathogenic variants have been described, including two relatively frequent susceptibility variants, c.511C > T and c.625G > A in the *ACADS* gene.

Over the past twenty years, diagnosis of fatty acid β-oxidation disorders in Slovakia has been done by selective metabolic screening, with the known limitations of the gas chromatography-mass spectrometry (GC/MS) technique and other additional methods. The elevated excretion of EMA, a metabolite closely associated with SCADD, was the most frequently observed abnormality in urinary organic acids. Since 2013, molecular genetic diagnosis of fatty acid β-oxidation disorders has been narrowly coupled with the extended newborn screening programme performed by the National Newborn Screening (NBS) Centre, which also includes monitoring of acylcarnitines by liquid chromatography-tandem mass spectrometry (LC-MS/MS). In accordance with the practice in many other countries, SCADD is not included in the obligatory panel of screened disorders in the full-area NBS programme in Slovakia because it does not meet the criteria for inclusion into the screening programme. However, when the upper limit of C4-acylcarnitine in a dried blood spot is exceeded, a suspicion of SCADD should be expressed. Moreover, the GC/MS analysis of organic acids in urine samples represents a part of the complex examination for confirmation or exclusion of suspected metabolic disorders, including SCADD.

Thus, SCADD has been biochemically defined as the presence of an increased level of C4-acylcarnitine in plasma and/or increased EMA excretion in urine under non-stressful conditions in at least two measured samples and genetically as the occurrence of biallelic pathogenic variants or one pathogenic variant in the *trans* position, with susceptibility variants c.625G>A or c.511C>T in the *ACADS* gene.

The elevation of C4-acylcarnitine represents the most frequent abnormality detected in the NBS in Slovakia. After elucidation of the molecular genetic causality of this biochemical phenomenon, the need for the application of a routine and low-cost scheme for SCADD diagnosis has logically arisen.

## Methods

### Patients included in the study

This study was conducted on sixty-two individuals with biochemical findings suggestive of SCADD, characterised by elevated C4-acylcarnitine concentrations in plasma and/or elevated EMA concentrations in urine under non-stressed conditions on at least two occasions [6]. Probands were chosen on the basis of an exceeded cut-off limit for C4-acylcarnitine from the NBS. However, a few of them were patients registered in the regional metabolic centres because of suspected SCADD based on elevated urine EMA values, with or without clinical findings. These patients did not undergo NBS included acylcarnitine examination. In the past, urine, serum and dried blood spots or peripheral uncoagulated whole blood samples for DNA and biochemical analyses were subsequently provided by these metabolic centres. In the majority of the selected probands, all appropriate data (C4-acylcarnitine and/or EMA level) were available. However, clinical data were available only for a few patients. Informed consent was obtained from all parents (or legal representatives) of children included in the study.

### Control groups

We used three control groups in this study to evaluate and compare the biochemical and molecular genetic findings in our cohort with data acquired from the common Slovak population. Informed consent was obtained from all individuals or from the parents (or legal representatives) of children included in the study. Control group A consisted of fifty randomly selected individuals with unknown ethnicity never suspected for SCADD and was created with the aim of estimating the frequency of two common susceptibility variants and two rare pathogenic variants in the Slovak population. Control group B consisted of ninety random newborns from NBS with C4-acylcarnitine parameters within the reference range and was used to compare the C4-acylcarnitine values with those measured in our cohort of the SCAD-deficient patients. Control group C consisted of fifty-two children with physiological EMA urine excretion measured in our department and was created to compare EMA values with those measured in the cohort of the SCAD-deficient patients.

### Biochemical analyses

Metabolite analyses were performed in the NBS Centre, in the Department of Molecular and Biochemical Genetics of University Hospital, and the Department of Laboratory Medicine of University Children’s Hospital.

The acylcarnitine values were determined in the NBS Centre of Slovakia by a MassChrom® Amino Acids and Acylcarnitines from Dried Blood - LC-MS/MS (Chromsystems) enabling a very fast and reproducible quantification of the amino acids and acylcarnitines. Suspected values were those that exceeded the reference range of C4-acylcarnitine (cut-off limit > 0.95 μmol.l^− 1^) and ratios set by the Centre.

The urinary organic acids were determined by gas chromatography/mass spectrometry (GC/MS) analysis using the ITQ 1100™ Ion Trap Mass Spectrometer (Thermo Scientific) with a 30 m DB-XLB column with 0.25 i.d. (Agilent Technologies) and a constant helium column flow of 1.0 ml/min for 28 min. The methodology is based on the repeated extraction of organic acids by ethylacetate after mixing with 30 nmol of the 4-phenylbutyric acid (internal standard) and derivatisation by a mixture of BSTFA/TMCS/acetonitrile/pyridine with a ratio of 10:5:1:1 at 60 °C for 60 min. Quantification of organic acids was performed by comparison to pure standards and measured values were corrected on the recovery of added internal standard. Four points calibration curves were constructed that consisted of different amounts of the standards. All measured values of urinary organic acids were expressed as μmol/mmol creatinine and were compared with the reference data published by Blau et al. [8] and the Urine Metabolome Database (www.urinemetabolome.ca). A given reference interval for EMA urine excretion (0.1–13.9 μmol/mmol creatinine) was determined by the Department of Molecular and Biochemical Genetics.

### Molecular-genetic analyses

#### DNA extraction, amplification and sequence analysis

Genomic DNA was extracted from peripheral blood leukocytes using NucleoSpin® Blood (Macherey-Nagel) according to the manufacturer’s protocol. Applying the recommendation from GeneReviews® for the molecular genetic algorithm for SCADD diagnosis, we primarily focused our study on two susceptibility variants, c.625G>A and c.511C>T, and one missense pathogenic variant, c.319C>T [6]. We decided to perform sequence analyses because relevant variants were found only in three biochemically defined SCADD individuals. The entire coding sequence of the *ACADS* gene was amplified by polymerase chain reaction (PCR) using 10 pairs of primers designed by our laboratory using the Primer3 software. Purified PCR products were sequenced using the ABI 3100® Avant Genetic Analyzer (Applied Biosystems) using a BigDye® Terminator v3.1 Cycle Sequencing Kit (Applied Biosystems). Mutation analysis was performed using SeqScape® Software v2.6. All identified sequence variants were confirmed by sequencing of both DNA strands.

### Algorithm for molecular genetic screening

Based on our experience with the findings of frequent variants in the Slovak population, we performed targeted analyses of four coding *ACADS* regions as a routine molecular genetic algorithm. The optimised reaction cycling conditions for PCR amplification of four exons are shown in Table 1.

**Table 1:** Primer sequences and PCR conditions

*ACADS* variant	Primer sequence (5ʼ → 3ʼ)	PCR conditions	PCR product (bp)
c.310_312 delGAG (exon 3)	for: TCACATGGCCTGAGTTTCTG rev: GGCCTACCCAGTAGGACCA	95 °C 2 min, (95 °C 1 min, 59.5 °C 1 min, 72 °C 1 min) 30×, 72 °C 5 min	396 bp
c.511C > T (exon 5)	for: CTGGTGCCCTTAGGTTGTGT rev: TTGCCCCAGAGCAAAAATAG	95 °C 3 min, (95 °C 30 s, 55 °C 30 s, 72 °C 45 s) 35×, 72 °C 10 min	319 bp
c.625G > A (exon 6)	for: CTGAGCTTCTGAGGGAGGTG rev: ATGTCCAGGGTTTGCTGTG	95 °C 15 min, (95 °C 20 s, 60 °C 30 s, 72 °C 1 min) 30×, 72 °C 5 min	375 bp
c.1138C > T (exon 10)	for: GCCCCTTCTCCAGCTTTC rev: AGTCTCTGAGCCGGGGTTG	95 °C 2 min, (95 °C 1 min, 59.5 °C 1 min, 72 °C 1 min) 30×, 72 °C 5 min	294 bp

Under the current algorithm, four frequent variants were screened using restriction fragment length polymorphism of PCR amplified fragments analysis (PCR-RFLP) as a quick and cost effective alternative in comparison with the sequence analysis. PCR-amplified products were digested by specific restriction endonucleases, and the restriction fragments yielding typical restriction patterns were then visualised on a 2.5% agarose gel (Serva) or a 3.5% metaphor agarose gel (Lonza, Table 2).

**Table 2:** Restriction endonucleases that were used with their palindromic recognition sequences and resulting restriction patterns

*ACADS* variant	Restriction endonuclease (recognition sequence)	Restriction pattern
c.310_312delGAG	*BseRI* (GAGGAG(N)10^)	51 bp + 168 bp + 177 bp (wt)/ 177 bp + 219 bp (mut)
c.511C>T	*HpaII* (C^CGG)	98 bp + 141 bp + 80 bp (wt)/ 239 bp + 80 bp (mut)
c.625G>A	*Hpy188I* (TCN^GA)	71 bp + 294 bp + 10 bp (wt)/ 71 bp + 26 bp + 268 bp + 10 bp (mut)
c.1138C>T	*BsrBI* (CCG^CTC)	115 bp + 179 bp (wt)/ 294 bp (mut)

### Statistical analysis

To evaluate the biochemical findings revealed in SCAD-deficient patients, we used data from two control groups without clinical and/or biochemical suspicion for SCADD. Statistical tools were used to determine the statistically significant differences in the C4-acylcarnitine and the EMA excretion between the patients and the control groups. A single-factor analysis of variance (ANOVA) compared the means between the studied groups and determined whether there were significant differences. Subsequently, we performed a correlation analysis by separating the C4-acylcarnitine and EMA values according to the SCADD genotypes and compared those with each other as well as with the control groups (Table 4, Table 5, Fig. 2, and Fig. 3).

## Results

In the present study, we evaluated the selected data from the cohort of sixty-two individuals with biochemical findings related to SCADD. The members of the cohort were selected according to the biochemical criteria typical for SCADD, which includes the overstepping of the stated limit of positivity (cut-off) for C4-acylcarnitine in a dried blood spot and the limit for EMA in urinary excretion. Based on these conditions, which had been met by probands in our cohort, we decided to perform a molecular genetic analysis of the *ACADS* gene.

**Fig. 1:**
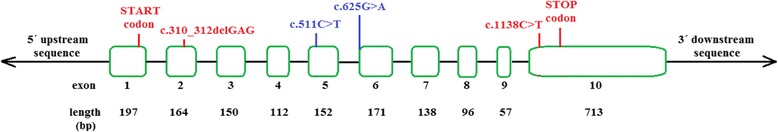
The structure of the *ACADS* gene and the location of the frequent sequence variants. A schematic representation of the two frequent susceptibility variants c.511C>T and c.625G>A (blue), two pathogenic variants c.310_312delGAG and c.1138C>T (red), and their location in the coding sequence (green) of the *ACADS* gene

In the initial study, according to the GeneReviews® recommendation, the detection of two common susceptibility variants and one pathogenic variant c.319C>T (exon 3) was applied [6]. The first susceptibility variant was a substitution c.625G>A (p.Gly209Ser, rs1799958) in exon 6, and the second susceptibility variant was a less frequent substitution c.511C>T (p.Arg171Trp, rs1800556) in exon 5. In the first forty-six individuals, pathogenic variant c.319C>T was not found in any allele, c.625G>A variant was present in thirteen alleles of which two probands were homozygous, and c.511C>T variant was present in one allele only. A discrepancy in the biochemical markers for SCADD (C4-acylcarnitine and EMA) and low frequency of the detected variants encouraged us to search for the occurrence of different pathogenic variants in the *ACADS* gene using sequence analysis of the entire coding region of the *ACADS* gene. To detect unknown variants, we selected nine patients with clear-cut biochemical parameters suggesting SCADD. Ethnicity was not considered as a criterion. First, the most remarkable finding was represented by a 3-bp deletion c.310_312delGAG (rs387906308) located in exon 3 of the *ACADS* gene (Fig. 1), resulting in a loss of a glutamine residue in amino acid position 104 in the precursor peptide (p.Glu104del). This change was detected in twelve alleles. The second important finding was a missense pathogenic substitution c.1138C>T (rs28940875) located in exon 10 of the *ACADS* gene (Fig. 1), causing a substitution of arginine to tryptophane at amino acid position 380 in the precursor peptide (p.Arg380Trp), which was detected in three alleles. According to MutationTaster, which is a software that performs in silico tests to estimate the impact of sequence variants on the gene product, both variants were disease causing, and in a causal link to SCADD. In the Genome Aggregation Database (gnomAD), the total allele frequencies for c.310_312delGAG and c.1138C>T are 1.81e-5 and 9.113e-5, respectively. Based on the acquired results, we proposed a routine molecular genetic algorithm for SCADD-suspected individuals, which included the detection of two rare pathogenic variants c.310_312delGAG and c.1138C>T simultaneously with the detection of two common susceptibility variants c.625G>A and c.511C>T, using PCR-RFLP analysis as a less expensive method compared to Sanger sequencing.

Next, we performed a retrospective molecular genetic analysis of previously admitted samples of newborns with elevated C4-acylcarnitine levels in NBS and individuals with clinical symptoms associated with SCADD and increased EMA urinary excretion. In six probands with one or two detected susceptibility variants but no pathogenic variant, extended screening of the *ACADS* gene by sequence analysis was not performed. The available data showed only slightly increased levels of EMA (Table 5), and thus, further examination has not been recommended.

In the following part of the study, we evaluated patients with supposed SCADD, comparing their clinical, biochemical and molecular genetic data. According to the established and generally accepted criteria for SCADD, we genetically confirmed fifty-six patients among sixty-two individuals initially included in this study. Allelic frequencies were 64% for c.310_312delGAG, 31% for c.1138C>T and 5% for the c.625G>A variant. Because these individuals met the molecular genetic criteria for SCADD (the presence of two pathogenic variants or one pathogenic variant in combination with the susceptibility variant), further sequence analyses of the entire *ACADS* gene were not considered necessary.

Among all 124 initially investigated alleles, 58.1% carried a c.310_312delGAG deletion, 28.2% carried a c.1138C>T pathogenic variant, 11.3% carried a c.625G>A variant, and 0.8% carried a c.511C>T variant. Only 1.6% comprised wild type alleles. The recognised SCADD genotypes accounted for twenty-four c.310_312delGAG homozygous probands, nineteen c.310_312delGAG/c.1138C>T compound heterozygotes, eight c.1138C>T homozygotes, and five c.310_312delGAG/c.625G>A compound heterozygotes. Based on the available data, most of these SCAD-deficient individuals (85.7%) were of Roma ethnicity. In this cohort, only 1.8% of studied persons were Caucasian. The ethnicity of 12.5% of individuals was not known.

To evaluate the frequency of the four abovementioned sequence variants in the common Slovak population, we used control group A, which was comprised of fifty random individuals with missing data about their ethnicity. In this cohort of one hundred alleles, we found 25% c.625G>A alleles (nineteen in the heterozygous state and three in the homozygous state), and the c.511C>T allele was detected in four individuals in the heterozygous state, representing an allele frequency of 4%. Only one c.310_312delGAG allele in the heterozygous state was found in one individual (1%), and no allele carried the c.1138C>T pathogenic variant. Up to 70% of the individuals comprised wild type alleles (Table 3). The frequencies of susceptibility variants c.625G>A and c.511C>T were in accordance with data for other European Caucasian populations at 25% and 4%, respectively [9].

**Table 3:** The percentile representation of investigated genetic variants in the cohort of patients suspected of SCADD in comparison with control group A

Genotype	Wild type	c.511C>T	c.625G>A	c.310_312delGAG	c.1138C>T
SCADD patients (*n* = 62)	1.6%	0.8%	11.3%	58.1%	28.2%
Control group A (*n* = 50)	70.0%	4.0%	25.0%	1.0%	–

**Fig. 2:**
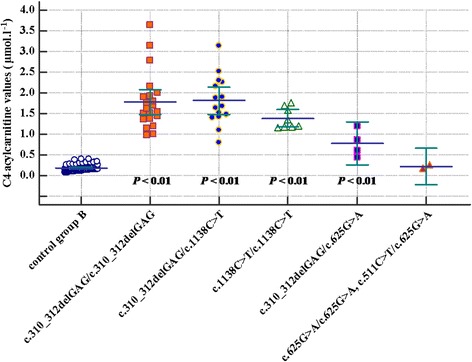
A multiple variables graph (MedCalc® v15.11.4) showing C4-acylcarnitine values (μmol.l^− 1^) in control group B compared with C4-acylcarnitine values sorted according to SCADD genotype and statistical significance related to the control group

**Fig. 3:**
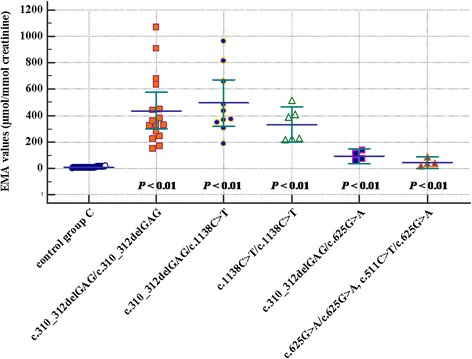
A multiple variables graph (MedCalc® v15.11.4) showing EMA values (μmol/mmol creatinine) in control group C compared with EMA values sorted according to SCADD genotype and statistical significance related to the control group

In a follow-up of our study, we compared C4-acylcarnitine values in ninety control individuals from the NBS (control group B) with C4-acylcarnitine values available in forty-eight of our fifty-six patients, with an apparently significant difference (Table 4). Similarly, we compared the available values of EMA excretion in thirty-six SCADD patients with fifty-two control urine samples (control group C) with analogical relations (Table 4). Subsequently, we sorted C4-acylcarnitine and EMA values measured in our cohort according to genotype (Table 5, Fig. 2, and Fig. 3). By comparing C4-acylcarnitine values in each genotype with the control group B, we observed significant differences in all cases (two probands with two susceptibility variants were not statistically evaluated). The EMA values showed significant differences in all genotypes compared to control group C.

**Table 4:** The comparison of C4-acylcarnitine and/or EMA values in the cohort of SCAD-deficient patients and control groups B and C, respectively

C4-acylcarnitine (μmol.l^− 1^)	control group B (*n* = 90)	patients (*n* = 48)
mean value ± SD	0.19 ± 0.07	1.64 ± 0.64^*^
minimal/maximal value	0.07/0.40	0.44/3.63
EMA (μmol/mmol creatinine)	control group C (*n* = 52)	patients (*n* = 36)
mean value ± SD	6.96 ± 3.44	396 ± 249^*^
minimal/maximal value	0.84/15.96	54/1070

Data are shown as the mean ± SD

^*^statistical significance *P* < 0.01

**Table 5:** The comparison of C4-acylcarnitine and EMA values in the cohort of SCAD-deficient patients sorted according to genotype

Genotype		c.310_312delGAG/c.310_312delGAG	c.310_312delGAG/c.1138C>T	c.1138C>T/c.1138C>T	c.310_312delGAG/c.625G>A	c.625G>A/ c.625G>A and c.511C > T/ c.625G>A
C4-acyl-carnitine (μmol.l^−1^)	number of patients	*n* = 21	*n* = 15	*n* = 8	*n* = 4	*n* = 2
	mean value ± SD	1.75 ± 0.70	1.78 ± 0.63	1.29 ± 0.26	0.78 ± 0.33	0.18; 0.25
	minimal/ maximal value	0.97/3.63	0.80/3.14	1.02/1.75	0.44/1.20	–
EMA (μmol/mmol creatinine)	number of patients	*n* = 16	*n* = 10	*n* = 6	*n* = 4	*n* = 4
	mean value ± SD	435 ± 262	494 ± 244	330 ± 126	93 ± 37	45 ± 28
	minimal/ maximal value	146/1070	188/965	216/514	54/135	20/85

Data are shown as the mean ± SD (except for c.625G>A/c.625G>A and c.511C > T/c.625G>A genotypes in two patients, which individual values for C4-acylcarnitine are given)

C4-acylcarnitine reference values according to NBS of Slovakia are 0–0.95 μmol.l^−1^

EMA reference values according to the Department of Molecular and Biochemical Genetics range from 0.1–13.9 μmol/mmol creatinine

Statistical analysis of SCADD genotypes with each other showed a significant difference only between the most frequent genotype c.310_312delGAG/c.310_312delGAG and c.310_312delGAG/c.625G > A, c.625G > A/c.625G > A, and c.511C > T/c.625G > A genotypes.

Because the majority of SCAD-deficient patients in our cohort were from NBS and asymptomatic, in these cases, a genetic examination was indicated on the basis of the exceeded cut-off limit for C4-acylcarnitine. Detailed information about the health status of 20 patients with a determined SCADD genotype (pathogenic variant/pathogenic variant, pathogenic variant/susceptibility variant) was at our disposal. Two patients with another confirmed severe diagnosis (MCADD and mitochondrial ATP synthase deficiency caused by mutations in the *ACADM* and *TMEM70* genes, respectively) were excluded from this twenty-member cohort because of the impossibility of distinguishing the effect of these two clinical units. The eighteen remaining patients (32% of the probands with genetically confirmed SCADD) manifested a broad range of symptoms, including developmental delay and intellectual disability present in ten patients, tonus disorder in six patients, metabolic acidosis in five patients, seizures/epilepsy in four patients, hepatopathy and hypoglycaemia in three patients and various symptoms in eight patients (Table 6). In eight patients, a variety of sporadic, non-specific or possibly unrelated symptoms were present. Furthermore, in the family history of two asymptomatic patients, there were two cases of sudden infant death syndrome (SIDS), and in one patient, the diagnosis of MCADD, often associated with SIDS, was confirmed.

**Table 6:** Eighteen symptomatic SCAD-deficient patients included in the present study with data concerning ethnicity, biochemical findings, clinical phenotype and *ACADS* genotype

Patient No.	Ethnicity	Biochemical findings					Phenotype	*ACADS* genotype
		C4^a^	C4/C2^a^	C4/C3^a^	C4/C8^a^	EMA^b^		
1	R	1.53	0.21	1.35	40.38	N/A	microcephaly, DD/ID, marked hypotonus, horizontal nystagmus	c.310_312delGAG/c.310_312delGAG
9	R	N/A	N/A	N/A	N/A	634	DD/ID, apnoic pause, bleeding into the adrenal glands in the neonatal age, hypoglycaemia	
10	R	1.44	0.05	0.81	30.64	363	central hypotonus with acral hypertonus, laryngomalatia	
12	R	1.07	0.07	1.07	40.75	146	epilepsy, hypotonus, irritability, cerebral palsy, pectus carinatum, kyphoscoliosis	
14	R	0.97	0.08	0.61	59.6	1070	recurrent sepsis, MAC, hepatosplenomegaly, severe hepatopathy along with gastroenteritis	
15	C	2.77	0.11	3.26	60.23	169	mild MAC in neonatal age, at age 2 yo - DD/ID, hypotonus, at age 3 yo – without DD/ID	
17	R	N/A	N/A	N/A	N/A	329	severe DD/ID, parents consanguinity, MAC, brother – DD/ID with no biochemical positivity to SCADD	
23	R	1.19	0.07	0.57	129	322	hypoglycaemia, elevated CK	
24	R	1.00	0.10	0.53	60.88	N/A	mild MAC	
25	R	1.90	0.14	1.98	52.34	657	DD	c.310_312delGAG/c.1138C>T
32	R	N/A	N/A	N/A	N/A	306	prematurity, severe DD/ID, quadruparesis, recurrent hypoglycaemia	
33	R	N/A	N/A	N/A	N/A	965	DD/ID (milder than brother - patient No. 32)	
35	R	1.52	0.08	0.94	77.08	433	mild hepatomegaly, hyperammonemia, MAC	
52	N/A	0.61	0.06	5.28	10.18	N/A	DD/ID ^c^	c.310_312delGAG/c.625G>A
53	R	1.20	0.07	0.72	17.93	135	short stature, ophtalmoplegia (first sign – suspicion for congenital myasthenia), hypomimic facies, severe kyphoscoliosis, DD/ID, cachexia, hypotonus, respiratory failure, diffuse brain edema, coma vigile (high suspicion for other unknown neuromuscular disease)	
54	R	N/A	N/A	N/A	N/A	71	epilepsy	
55	R	0.44	0.03	0.19	50.42	54	recidivating granulomatous hepatopathy with hepatomegaly, mild hypertonus, mild DD/ID, epilepsy, apnoic pauses	
56	R	0.86	0.03	0.41	21.15	110	recurrent seizures	

*R* Roma, *C* Caucasian, *N/A* not available

^a^reference values - C4-acylcarnitine 0–0.95 μmol.l^−1^; C4/C2 0–0.04; C4/C3 0.04–0.5; C4/C8 0.8–15 (values specified in the Slovak NBS Centre by MassChrom® Amino Acids and Acylcarnitines from Dried Blood - LC-MS/MS (Chromsystems)

^b^EMA reference values according to Department of Molecular and Biochemical Genetics are in the interval 0.1–13.9 μmol/mmol creatinine

^c^recommended repeated biochemical analysis to meet metabolic criteria for SCADD

## Discussion

In Slovakia, a newborn screening programme was introduced in 1972 by screening phenylketonuria (PKU) and was later broadened to four diseases. Since 2013, the spectrum of the NBS panel has been extended to thirteen current disorders. LC-MS/MS analysis has been applied for the quantitative measurement of acylcarnitines and amino acids. The implementation of this technology has proven to be of great value, especially in the diagnosis of β-oxidation disorders.

A marked elevation of EMA in the urine without other relevant metabolic changes was detected relatively frequently in the past, mostly in patients from the northeast regions of the country. However, after initial molecular genetic analyses, no significant increase in the frequency of the *ACADS* sequence variants typical for the European population and causing elevation of the specific biochemical parameters C4-acylcarnitine and EMA were observed in the screened patients. Because this discrepancy could point to the occurrence of unknown or rare variants in the Slovak population, we decided to perform a sequence analysis of all ten exons of the *ACADS* gene. The crucial finding consisted of a high occurrence of two rare variants in the probands of Roma ethnicity. The first variant was a 3-bp deletion c.310_312delGAG in exon 3, and the second was a missense variant c.1138C>T in exon 10, both described by Corydon et al. [10] and considered as being pathogenic. Based on these unusual preliminary results, the proposal for an alternative molecular genetic algorithm was announced by Chandoga et al. [11]. At present, all SCADD-suspected individuals in Slovakia are diagnosed according to the current algorithm, including the detection of the two pathogenic variants c.310_312delGAG and c.1138C>T, in combination with a search for the two susceptibility variants c.625G>A and c.511C>T.

Generally, a Roma ethnic group represents a cluster of genetically isolated founder populations with a spectrum of frequent Mendelian disorders, as a consequence of a high rate of consanguinity associated with the occurrence of founder mutations. Thus, their genetic background differentiates them from the majority ethnic group in Slovakia, resulting in an increased incidence of phenylketonuria, fatty acid oxidation disorders (MCAD deficiency), primary congenital glaucoma, and monogenic hearing impairments and a slightly higher occurrence of congenital hypothyroidism. An isolated ATP synthase deficiency due to the pathogenic variant 317-2A>G in the *TMEM70* gene and congenital myasthenic syndrome due to the deletion of 1267delG in the *CHRNE* gene was referred to later [12, 13]. In contrast, cystic fibrosis and congenital adrenal hyperplasia did not occur in this group [14, 15]. Interestingly, the cut-off limit for immunoreactive trypsinogen (IRT) in the screening of cystic fibrosis differentiates substantially between the Slovak people of Caucasian origin and the Roma ethnic group, in which the level of IRT is significantly elevated in as many as 32% of newborns. This fact has led to the standard identification of the ethnicity in newborn screening and the introduction of different cut-off limits in Roma newborns [15].

As ethnicity is required information included in the data of each newborn and is disclosed by NBS, we found that almost 86% of our SCAD-deficient individuals belong to the Roma ethnic group, and 2% are Caucasian. The ethnicity of approximately 12% of patients from our cohort was not proclaimed. After evaluation of the data obtained in this study and screening the data related to metabolic disorders, we were able to state that SCADD is the most frequent metabolic disturbance in Slovakia, with an especially high frequency in the Roma ethnic group. This fact is likely due to the founder effect and/or the fact that the Slovak Roma ethnic group has the highest rate of consanguinity in Europe [16]. Thanks to data received from NBS collected during 2016 from 57.657 investigated newborns, approximately 84.5% are Caucasian and 15.5% belong to the Roma ethnic group. The established C4-acylcarnitine cut-off limit by the NBS exceeded 0.01% in Caucasian newborns, compared to 0.99% of Roma newborns.

Metabolic changes in blood acylcarnitines (elevated C4-acylcarnitine) are well correlated with the urine metabolite typical for SCADD (elevated EMA). To evaluate the range of the biochemical abnormalities typical for SCADD, we assembled two control groups of healthy probands and compared blood C4-acylcarnitine and urine EMA excretion with the same parameters in SCADD patients from our cohort. After creating subgroups of SCADD patients according to the different genotypes, we observed a significant difference between genotypes harbouring two pathogenic variants with markedly higher levels of both metabolites, compared to genotypes comprising of one pathogenic variant in combination with one susceptibility variant or only two susceptibility variants.

Despite the fact that an asymptomatic outcome is prevalent, two main clinical phenotypes of SCADD have been described. The first form is presented in infants as acute acidosis and muscle weakness, and the second form is presented in middle age as chronic myopathy. Patients with neonatal onset have a variable clinical manifestation including metabolic acidosis, failure to thrive with poor feeding, developmental delay, hypotonus and myopathy [17]. In sporadic cases, dysmorphic facial features, vomiting, hepatic dysfunction after premature delivery and bilateral optic atrophy were described [18]. In the biochemical phenotype, the episodes of metabolic decompensation were characterised by ketotic hypoglycaemia, metabolic acidosis and/or hyperammonaemia [6, 19]. However, SCADD is manifested predominantly as neurological symptoms, due to the chronic central nervous system toxicity associated with the metabolites that are accumulated, ethylmalonic acid (EMA) and butyric acid. EMA inhibition of cerebral creatine kinase activity and the constituents of the respiratory chain are among the crucial pathobiochemic effects [20]. This impact on the brain may reflect the fact that short-chain monocarboxylic acids cross the blood-brain barrier more easily than longer-chain acids [21].

Our findings concerning the high frequency of SCADD in Slovakia opens several considerations. At present, since the majority of SCADD patients were asymptomatic newborns detected because of expanded NBS, we were not able to fully explore the clinical significance and consequences of frequent c.310_312delGAG and c.1138C>T variants in terms of morbidity and mortality. To assess a possible clinical outcome, we evaluated the present symptoms in eighteen SCADD patients with no other confirmed diagnoses. The most prevalent signs were in concordance with the literature and included developmental delay, intellectual disability, tonus disorder, metabolic acidosis, seizures/epilepsy, hepatopathy, and hypoglycaemia. Moreover, most of these symptomatic patients were, at the time of the molecular genetic diagnosis, older than two months. Thus, it is important to observe these patients and provide them with preventive dietary recommendations.

The literature and our findings show that the interpretation of the acquired molecular genetic data still remains problematic, mainly in the case of common variant/variant genotype combinations. On the basis of expression studies, two susceptibility variants, c.625G>A and c.511C>T, represent the disease-predisposing variants, which are insufficient for SCADD manifestation. They remain catalytically active but cause a higher thermo-lability of enzyme and an elevated excretion of ethylmalonic acid in carriers [22]. Thus, there must exist other unidentified additional factors (environmental, cellular and/or genetic) that are likely needed for the reduction of residual enzyme catalytic activity of susceptibility variants below a critical threshold and that are responsible for the expression of the disease [10]. The biallelic presence of two susceptibility variants may be associated with an asymptomatic condition, representing only certain biochemical characteristics [23]. In the case of positive biochemical and/or clinical findings, it would be necessary to search for and carry out a mutational analysis of the entire *ACADS* coding sequence and/or seek an alternative diagnosis. The spectrum of clinical manifestation is broad and may be non-specific and thus problematic for evaluation.

Screening for SCADD has now been included in the expanded newborn screening programmes in most of the U.S. and the Australian territories. However, the relevance of early diagnosis of SCADD did not show a clinical usefulness [24]. Accordingly, doubts over including SCADD in the NBS programme were expressed by the Newborn Screening Expert Group of the American College of Medical Genetics on the basis of the lack of the specific treatment and a poorly understood natural history of the disorder [5]. The most fundamental criteria for NBS include that the screened disease or condition should be serious or potentially serious, should be relatively common, and must be possible to differentiate diseased from non-diseased individuals. The following queries warrant further investigation: the seriousness of the metabolic disturbance caused by two abovementioned pathogenic variants, the estimated prevalence of SCADD in the Slovak population and its relation to ethnicity, symptoms that may be specific to SCADD, and if the manifestation or other health handicaps can be prevented by appropriate diet or treatment. For these questions to be answered, a long-term full-area study will be required in the future. Although SCADD is characterised by a lack of clinical significance and by the impossibility to differentiate affected and non-affected individuals in most cases, it is important to detect this disorder before a severe and/or possible life-threatening manifestation. Moreover, the genetic background as well as the social status of the patients should be taken into account. A clinical manifestation of the disease can be triggered by physiological stress, such as unsatisfactory eating habits and poor newborn care. It is important to emphasise that these stressful situations are difficult to manage by people living in poor social conditions, which were often reported in our patients.

SCADD treatment remains ambiguous because of the frequent asymptomatic stage of the disorder, and no generally accepted dietary recommendations exist. Despite the fact that the majority of patients are asymptomatic, the need for therapeutic intervention is especially questionable in patients with high levels of EMA. In our study, we observed a considerable difference in the EMA excretion, depending on genotype. The carriers of two pathogenic variants had excreted significantly higher levels of this metabolite. As the occurrence of the neurological symptoms in SCADD have been postulated to be related to EMA [25], therapy should be considered in these patients before the potential onset of symptoms.

In some patients, treatment included riboflavin (a precursor of FAD), a low fat/high carbohydrate diet and avoiding fasting. Seizures were treated with anticonvulsants (avoiding valproate). Because the risk of metabolic decompensation remains higher, it is important to be alert and avoid dehydration, metabolic acidosis and hypoglycaemia in the case of infectious illness. A manifestation of metabolic acidosis can be suppressed by promoting anabolism and provides an alternative source of energy by an intravenously supplied dextrose, with or without insulin [6]. There was an optimal prognosis in asymptomatic carriers of pathogenic variants. On the other hand, there are differing opinions within the medical community that SCADD does not represent a real disease and does not have clinical significance. Rhead [26] argued that homozygous inactivating variants lower butyrate oxidation/pathway flux and C4-CoA dehydrogenation by 40–60% in vivo only and that most patients are asymptomatic with otherwise non-specific clinical presentation, very often due to other confirmed causes.

In most cases, SCADD is detected during the perinatal period and many countries do not meet the basic criteria for inclusion in the full-area NBS; it is questionable how to interpret the specific data collected in this study. It remains to be determined if SCADD should be officially included in the NBS programme and what the benefits would be for newborn healthcare. In any case, the high frequency of this genetic metabolic abnormality in the Slovak population approves the justification for extending the NBS programme in the diagnosis of β-oxidation disorders and enables us to detect and consider one of the largest described groups of individuals with SCADD in the world. Additionally, it assists in subsequently monitoring biochemical phenotypes and conditions that trigger a clinical manifestation over a long period of time. A long-term prospective study is need to objectively search for possible or not yet referred phenotypes, morbidity, and mortality rate in children with a confirmed metabolic disturbance.

## Conclusion

Generally, SCADD represents a rare autosomal recessive inborn metabolic disorder of mitochondrial β-oxidation affecting from 1 in 35,000 to 1 in 50,000 newborns. In Slovakia, the suspicion for SCADD is expressed relatively frequently based on the elevated level of the specific biomarker C4-acylcarnitine, using LC-MS/MS as a part of the extended NBS programme. Molecular genetic analysis of the *ACADS* gene revealed the presence of two pathogenic variants with a high occurrence, especially in the Roma ethnic group. Only a few patients have clinical symptoms, indicating an ambiguous clinical significance of the detected sequence variants. According to recent data, the screening prevalence of SCADD in the Slovak majority ethnic group represents 1: 9.745. In the Roma ethnic group, it reaches 1:100, and thus, SCADD represents the most frequent metabolic abnormality in this population.

## Abbreviations

*ACADS*: Gene-coding short-chain acyl-CoA dehydrogenase
C4-acylcarnitine: Butyryl/isobutyrylcarnitine
C4-CoA: Butyryl-CoA
DD/ID: Developmental delay/intellectual disability
EMA: Ethylmalonic acid
GC/MS: Gas chromatography-mass spectrometry
IRT: Immunoreactive trypsinogen
LCHAD: Long-chain 3-hydroxyacyl-CoA dehydrogenase
LC-MS/MS: Liquid chromatography-tandem mass spectrometry
MAC: metabolic acidosis
MCAD: Medium-chain acyl-CoA dehydrogenase
MCADD: Medium-chain acyl-CoA dehydrogenase deficiency
NBS: Newborn screening
PCR-RFLP: Restriction fragment length polymorphism of PCR amplified fragments
SCAD: Short-chain acyl-CoA dehydrogenase
SCADD: Short-chain acyl-CoA dehydrogenase deficiency
SIDS: Sudden infant death syndrome
